# In vitro evaluation of cell viability and expression profile of growth factors in mouse Sertoli cells exposed to Delta-9-tetrahydrocannabinol: a mechanistic insight into the cannabinoid-induced testicular toxicity

**DOI:** 10.1186/s40360-023-00704-8

**Published:** 2023-11-09

**Authors:** Shadi Mohammadpour-Asl, Shiva Roshan-Milani, Amin Abdollahzade Fard, Ali Golchin

**Affiliations:** 1 Department of Physiology, School of Medicine, Urmia University of Medical Sciences, Urmia, Iran; 2 Cellular and Molecular Research Center, Cellular and Molecular Medicine Research Institute, Urmia University of Medical Sciences, Urmia, Iran; 3 Neurophysiology Research Center, Cellular and Molecular Medicine Research Institute, Urmia University of Medical Sciences, Urmia, Iran; 4 Nephrology and Kidney Transplant Research Center, Clinical Research Institute, Urmia University of Medical Sciences, Urmia, Iran; 5 Department of Clinical Biochemistry and Applied Cell Sciences, School of Medicine, Urmia University of Medical Sciences, Urmia, Iran

**Keywords:** Apoptosis, Endocannabinoid system, Growth factors, Male fertility, TM4, THC

## Abstract

The potentially adverse effects of cannabis (marijuana), a common leisure compound, on male reproductive performance are a reason for concern. δ-9-tetrahydrocannabinol (THC), the primary active component of marijuana alters testicular cells’ proliferation and function which affects male fertility and causes testicular cells dysfunction and apoptosis. The main objective of this study was to investigate the possible mechanism underlying the toxic effects of THC with a mechanistic insight into Sertoli cell-based reproductive dysfunction. The *Mus musculus* Sertoli cell line (TM4) was cultured and exposed to different concentrations of THC and, MTT (3-(4, 5-Dimethylthiazol-2-yl)-2,5-diphenyltetrazolium bromide) assay was then performed for evaluating cell viability. The expression of caspase-3 gene and genes related to growth factors were analyzed by real-time RT-PCR. Western blotting was performed for evaluating protein expression level. THC concentration-dependently decreased the TM4 viability with a significant effect starting at concentration of 1 μM and reaching about 75% of the control level at the concentration of 50 μM (IC_25_). Moreover, caspase-3 mRNA expression levels significantly increased while growth factors mRNA levels decreased in THC-exposed cells compared to unexposed cells. There was also a significant reduction in related protein levels in THC group. Administration of the THC promotes cytotoxic and apoptotic effects on TM4 cells partly through down-regulation of growth factors expression. Increased apoptosis, over expression of caspase-3, and down-regulation of growth factors expression in Sertoli cells exposed to THC may be a reflection of THC-induced testicular toxicity, which may be partly involved in infertility associated with marijuana smoking or medical cannabis use.

**Supplementary Information:**

The online version contains supplementary material available at 10.1186/s40360-023-00704-8.

## Introduction

Substance use among men of reproductive age remains a significant health concern, as that users of most addictive drugs show hypogonadism and impaired fertility. Young men account for a substantial subset of *Cannabis sativa* (marijuana) users and approximately 42% of young adults ages 19 to 30 reported marijuana use in the United States in the past year [1]. Emerging evidence has demonstrated that marijuana impairs male reproductive activity so that regular marijuana use has been linked to a lower semen quality and testosterone level [2–5] as well as a higher risk of testicular cancer [6, 7]. δ-9-tetrahydrocannabinol (THC), the major constituent and the primary active component of the marijuana, is a receptor agonist for the endocannabinoid system (ECS) and the most important exocannabinoid that has been studied [8]. The effects of THC on male fertility are still a topic of ongoing research. Some studies have reported no impact of THC exposure on sperm concentration or germ cell lineage in human [9] and mice [10] testis tissue. However, many other studies have shown that THC affects male fertility and causes gonadal dysfunction mainly at the testis and sperm levels. Accordingly, THC alters serum testosterone level and decreases sperm count, motility, normal morphology and acrosome reaction [2, 11–14]. Sperm DNA methylation [15], capacitation and transcripts levels [16] have also been altered by THC exposure. Exocannabinoids may also disrupt the physiological activity of endocannabinoids in male reproduction and so affect male fertility by interfering with the ECS and disrupting the ECS’s delicate balance [17]. In spite of these reports indicating that THC is involved in testicular toxicity and reproductive dysfunction, the underlying cellular and molecular mechanisms remain incomplete. Impaired spermatogenesis and irreversible infertility can result from alterations in Sertoli cell function and loss [18, 19]. Although toxicants, such as THC, are known to disrupt Sertoli cells, the detailed molecular events and underlying mechanisms involved in this process remain largely unknown. It has been suggested that THC-induced testicular toxicity may happen partly through induction of the early apoptosis of testicular germ cells and somatic cells [20–23]. Previous studies have demonstrated that THC induces apoptosis in different cells and tissues through various signaling pathways, including tumor necrosis factor-, p53-, oxidative stress-, and PI3K/Akt-dependent mechanisms. THC-induced apoptosis was also related to cytochrome c release and caspase-3 activation in cultured neurons and Sertoli cells [23, 24]. Several previous reports suggest that altered secretion of growth factors, as essential regulators in the process of cell life and development in the testis, are important contributing factors for several chronic conditions attributed to testicular toxicity and infertility. Vascular endothelial growth factor (VEGF) is one of the growth factors that alters testicular cells’ proliferation and metabolic activity [25]. It has been shown that both immature Leydig (TM3) and Sertoli (TM4) cells generate and secrete VEGF in the process of testicular function and spermatogenesis [26–28]. The removal of VEGFA isoforms causes subfertility and a reduction in the quantity of sperm in mice [29]. The glial cell-derived neurotrophic factor (GDNF) is also released by Sertoli and TM4 cells [30] and may increase stem cell numbers and sperm production [31]. Another common growth factor that is produced by Sertoli cells is the epidermal growth factor (EGF) which stimulates the proliferation of numerous cell types and appears to be involved in the formation of the testis and spermatogenesis [32, 33]. Fibroblast growth factor (FGF) is another Sertoli cell survival factor that plays a role in the proliferation and differentiation of testicular cells and spermatogenesis [34, 35]. Based on the previous reports, the production and secretion of growth factors and expression of their receptors can be impressed by cannabinoids in different cells and tissues [36–39]. It has been shown that doses of THC equivalent to those found in the serum of cannabis users inhibit proliferation of different cells by affecting several genes that encode for growth and apoptosis [40, 41]. To the best of our knowledge, there are no studies examining the effects of THC on testicular growth factors and their expression profile. Therefore, this study aimed to determine through which mechanism(s), an alteration of Sertoli cell death occurs as a result of THC exposure and investigate critical growth biomarkers that may link cannabinoid system components to apoptotic pathway activation. Therefore, to gain mechanistic insight into the THC-induced testicular toxicity, we examined the effect of THC on cultured mouse Sertoli cells and determined cell viability and apoptosis as well as expression profiles of a number of key testicular growth factors including VEGF, EGF, FGF and GDNF.

## Material and methods

### Cell culture

The *Mus musculus* Sertoli cell line, TM4 (purchased from Pasteur Institute, Tehran, Iran) was cultured in 25 cm^2^ tissue flasks, and grown in Dulbecco’s modified Eagle’s medium (DMEM)–F12/HEPES media (DNAbiotech, Iran) supplemented with 5% fetal bovine serum (Atocel, Austria), penicillin (50 units/mL), and streptomycin (50 units/mL). The flasks were kept in a cell incubator at 37 **°**C in a humidified environment with 95% O2 and 5% CO2. Every 24 hour, the medium was aspirated and replaced with fresh medium. The cultured cells were transferred and subcultured twice a week when they reached 70–80% confluence after brief trypsinization with 1% trypsin. Cell viability was assessed using trypan blue staining followed by hemocytometry, and 5× 10^4^–10^5^ cells were seeded in 25-cm^2^ flasks with 5 ml of new media. To test the effects of THC exposure on Sertoli cell viability, TM4 cells were seeded in a 96-well culture plate at a density of about 1 × 10^4^ cells/well and stabilized for 24 hours in each of the duplicate cultures. THC was purchased from (SERVA, USA) and utilized as the primary active component of the marijuana and an exogenous synthetic exocannabinoid. In the first phase of our study, we evaluated cell viability by examining a range of different concentrations of THC to determine the concentration-response relationship of THC and establish its toxicological thresholds. Accordingly, the cells were exposed to THC at the final concentrations of 0 (cells exposed to a THC-free media, as the control group), 0.1, 0.5, 1, 5, 10 and 50 μM for a duration incubation period of 24-h. The selection of THC concentrations and duration of exposure was based on previous studies that employed similar grouping strategies and suggested that this range of concentrations is more likely to produce toxic effects [23, 42, 43]. To reach the required concentrations, a 1 mM stock solution of THC was diluted in DMEM and fed to the cells. The medium was emptied from each well and replaced with 100 λ of THC-containing medium (6 wells for each concentration) or THC-free medium (6 wells as the control group). This process was carried out in two independent tests with duplicate cultures. The MTT (3-(4, 5-Dimethylthiazol-2-yl)-2,5-diphenyltetrazolium bromide) assay was then performed to investigate the cell viability of different concentration groups.

### Cell viability (MTT assay)

The MTT reagent powder (Sigma M5655-1G, Germany) was used to measure cell viability as previously described [44, 45]. MTT (5 mg/mL) stock solution in DMEM–F12 media was prepared and stored at 2–8 °C. Within 24-h, 100 μl of MTT dilution (1:10) was added to each well, and the cells were subsequently cultured in a CO2 incubator for a further 24-h. To dissolve the crystals, we used 100 μl of dimethyl sulfoxide (DMSO) for each well after a 3–4-hour incubation period. A 570 nm microplate reader was used to quantify the absorbance and optical density (O.D.). The following equation was used to determine the percentage of viable cells: O.D.570 of the treated sample (A) and O.D.570 of the control (B) were used to calculate the percentage of specific viability (A/B × 100). Analyses of the data produced mean and standard error of means (SEM), obtained from 2 determinations.

### Optimizing the cells and the concentration of THC for efficient molecular experiments

The authors were convinced that the concentration of THC that results in a 50% decrease in Sertoli cell viability (IC_50_) can induce a large amount of cell death, which influences the proper evaluation of growth factors. Based on this caveat, a moderate and sub-cytotoxic concentration of THC (IC_25_ = 50 μM, concentration inhibiting 25% of the cell growth) was considered for the rest of the molecular experiments. On the other hand, experiments have shown that dead cells settle slower than live cells, and this opens up the possibility to bleed out dead cells in a continuous centrifuge. Tests in a cell separator prove that this is feasible, and a significant portion of the dead cells can be removed from the system [46, 47]. Accordingly, after 24-h of THC treatment, the cells were trypsinized and centrifuged at 1500 rpm for 5 min so that nearly almost dead cells were in the supernatant and discarded. Cell pellets were used for RNA extraction.

### Real-time quantitative PCR

The expression of the caspase-3 gene as well as genes related to growth factors were investigated by real-time RT-PCR, using specific primers (Table 1). In this phase of the study, we utilized an IC_25_ of 50 μM (concentration inhibiting 25% of cell growth) obtained from the first phase of our study to investigate molecular aspects. For this purpose and as mentioned above, TM4 Sertoli cells were cultured and investigated in two groups including the “control” and “THC (50 μM)” groups. After 24-h of the last treatment, the cells were trypsinized and cell pellets were used for RNA extraction. According to the manufacturer’s protocol, total RNA was extracted using a Trizol reagent (Maxell, Iran). To eliminate genomic DNA nanodrop 2000, the collected RNA was processed with DNase I using the TURBO DNA-free kit (Invitrogen). Using a cDNA synthesis kit (pars tous, Iran) and 2 mL of total RNA, reverse transcription-PCR was done according to the manufacturer’s procedure. The MX300P was used to perform real-time PCR reactions using a Real Master Mix SYBR Green Kit (pcrbio, lot no: PB012619–110-1). The 2-duct method was used to figure out fold changes in gene expression as a ratio of the levels of expression in the THC-exposed group to the levels of expression in the control group. Expression of the β-actin gene was tested for internal control of the mRNA levels. Expression of the investigating genes was normalized to the endogenous control to acquire the relative threshold cycle (ΔCt) and related to the ΔCt of the control condition to find the relative expression level (2-ΔΔCt) of the THC group. mRNA levels of the investigating genes in the control cells were set at 1.00.

**Table 1:** Primer sequences used for qPCR

Gene	Forward sequence	Reverse sequence
VEGF	TGAACTTTCTGCTCTCTTGG	AACAAATGCTTTCTCCGCTC
EGF	GAAGCCCTCGTCACTGGTTG	TTCACGAATCCTTCCCGACA
FGF	GTGGGAGGAAGGGCGGTAAT	CCCCACCTCATCTCCTATCCA
GDNF	ATGAAGTTATGGGATGTCGTGGCT	GGGTCAGATACATCCACACCG
Caspase—3	GTTAACACGAGTGAGGATGTG	TACCCTGAAATGGGCTTGTGT
Beta actin	GGAGATTACTGCCCTGGCTCCTA	GACTCATCGTACTCCTGCTTGCTG

### Western blot examination

For protein extraction, a cell lysis buffer including protease and phosphatase inhibitors was utilized. The protein concentration in the cell lysates was determined using the Bradford reagent using bovine serum albumin (BSA) as the standard after cell lysis (Bio-Rad, TX). 50 μg of proteins were separated by 10% sodium dodecyl sulfate-polyacrylamide gel electrophoresis (SDS-PAGE) and then transferred to a polyvinylidene fluoride (PVDF) membrane. The membranes were blocked with 5% skim milk in TBS-T (0.1% Tris-buffered saline with Tween 20) at room temperature for 1 hour, then incubated with the main antibody and the secondary antibody, respectively (1:300 dilution). Using the enhanced chemiluminescence (ECL) western blot analysis kit, protein bands were observed (Amersham Pharmacia Biotech, Piscataway, NJ). The GDNF (sc-13,147), FGF (sc-55,520), and β-actin (sc-47,778) antibodies came from Santa Cruz Biotechnology Inc. and Elabscience Biotechnology Inc. in California, USA.

### Statistical analysis

The results are presented as mean ± SEM. SPSS 16 was used to perform statistical analyses with a significance level of *p* < 0.05. Normally distributed data (O.D. of MTT test) were analyzed using one-way parametric ANOVA followed by LSD post hoc test. The data related to gene and protein expressions, which were not normally distributed, were analyzed by the Mann-Whitney U test.

## Results

### THC-induced toxicity effects on Sertoli cell viability (MTT assay)

**Fig. 1:**
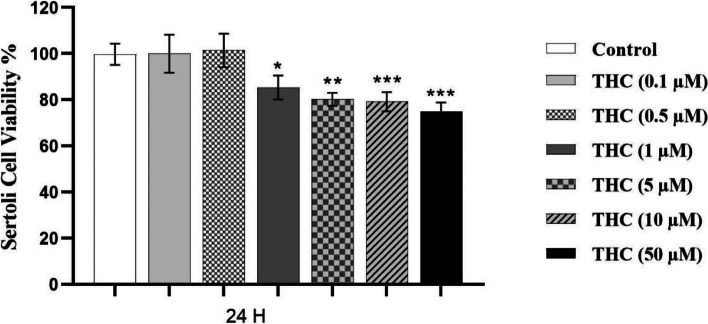
The effect of different concentrations of THC on TM4 cell viability. The effects of different concentrations of THC on TM4 cell viability measured by MTT assay following 24-h exposure period. Each bar represents the mean ± SEM, obtained from three independent experiments (*n* = 3). As the concentration of THC increased, cellular viability gradually decreased and reached approximately 75% of the control level at 50 μM of THC. Stars show the statistical significance of change among the groups. *p < 0.05, **p < 0.01, and ***p < 0.001 vs. control group (One–way ANOVA with LSD correction for multiple comparisons)

The viability of Sertoli cells after exposure to exogenous cannabinoid THC was assessed using MTT Assay. For that purpose, TM4 cells were exposed to THC at the final concentrations of 0 (cells treated with a THC-free medium, as the control group), 0.1, 0.5, 1, 5, 10, and 50 μM for 24-h, and the MTT assay was then used to determine cell viability. The MTT results indicated that the percentage of cell viability was reduced significantly with an increase in the concentration of THC. A more significant reduction compared to the control group was found in the group exposed to 50 μM THC (*P* < 0.001, Fig. 1). Accordingly, the cellular viability significantly decreased from 99 ± 4.6% in the control group to 74 ± 3.9% in the cells exposed to 50 μM THC, concentration which inhibited nearly 25% of the cell growth (*P* < 0.001, Fig. 1). Therefore these results showed that THC reduced cell viability of TM4 cells in a concentration-dependent manner with the IC_25_ value of 50 μM following a 24-h exposure period (Fig. 1).

### Effects of THC on mRNA expression of caspase-3 in Sertoli cells (real-time RT-PCR)

**Fig. 2:**
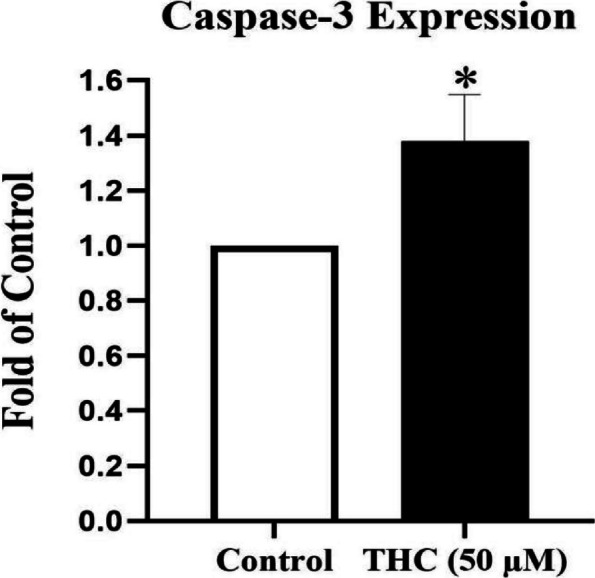
mRNA expression of caspase-3 in THC-exposed TM4 cells. The effect of THC (50 μM) for a duration exposure period of 24-h on caspase-3 gene expression level measured by RT-PCR in TM4 Sertoli cells. Caspase-3 mRNA expression was normalized to β-actin in each sample. THC significantly increased caspase-3 expression level. Data represent the mean ± SEM of three independent experiments (n = 3). Star shows the statistical significance of change between the groups. *p < 0.05 vs. control group (Mann–Whitney U-test)

Following the primary MTT experiments and analyses, an intermediate concentration of THC (50 μM, the IC_25_ value of THC) in a 24-h exposure period, was assumed for the next phases of the study. In order to confirm or modify the MTT results, quantitative real-time RT-PCR analysis of the mRNA levels of caspase-3 was performed, in the control and 50 μM THC exposed cells. The results showed that caspase-3 mRNA levels was significantly increased to 1.38 ± 0.17 in cells exposed to THC in comparison to the control cells (*P* < 0.05, Fig. 2).

### Effects of THC on mRNA expression of growth factors in Sertoli cells (real-time RT-PCR)

**Fig. 3:**
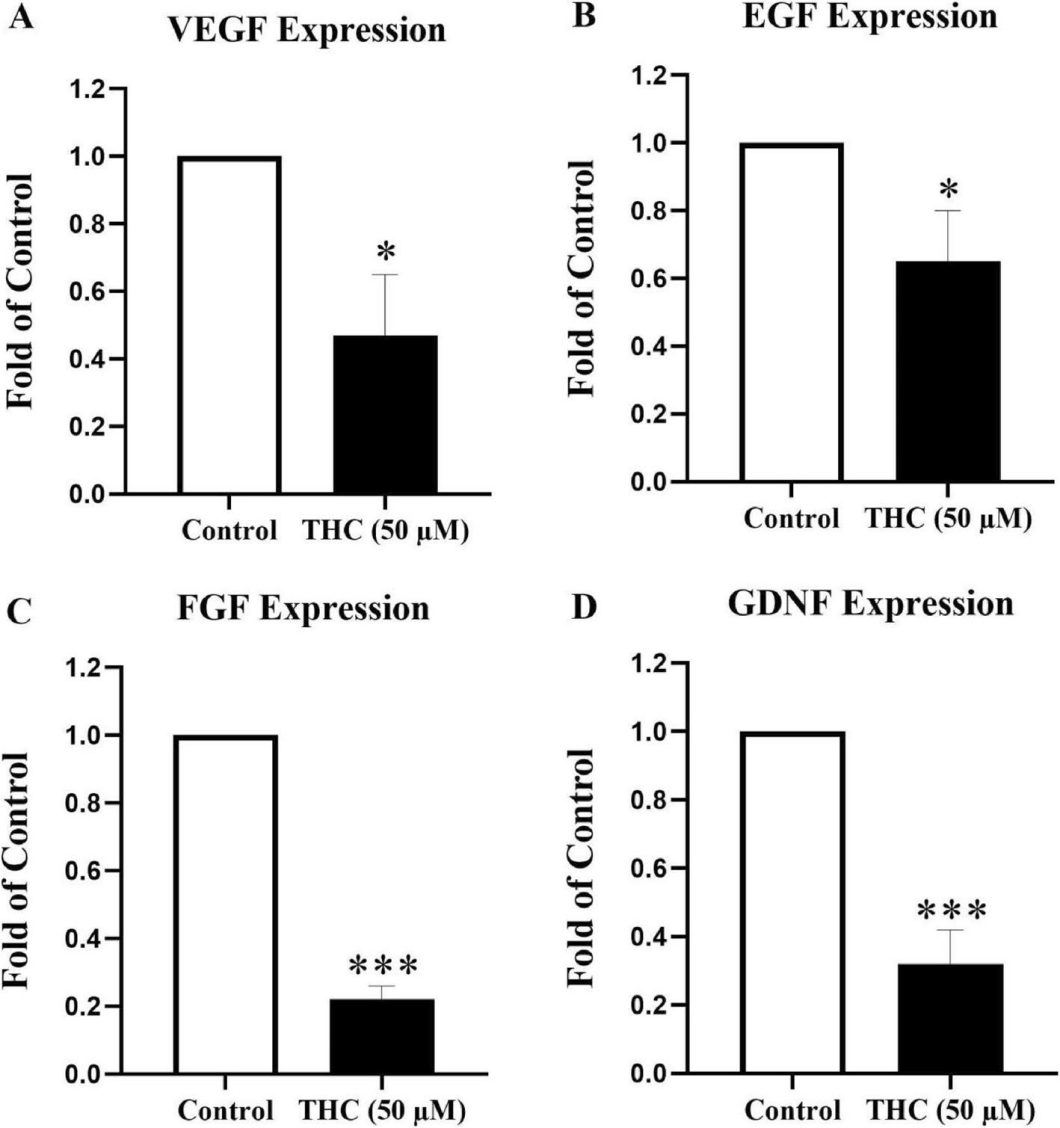
mRNA expression of growth factors genes in THC-exposed TM4 cells. The effect of THC (50 μM) for a duration exposure period of 24-h on gene expression level of a number of key testicular growth factors VEGF (**A**), EGF (**B**), FGF (**C**), and GDNF (**D**) measured by RT-PCR in TM4 Sertoli cells. mRNA expressions were normalized to β-actin in each sample. THC significantly decreased growth factors expression levels. Data represent the mean ± SEM of three independent experiments (*n* = 3). Stars show the statistical significance of change between the groups. *p < 0.05 and ***p < 0.001 vs. control groups (Mann–Whitney U-test)

Following above mentioned MTT and caspase-3 evaluations, the next series of experiments were performed to investigate the possible mechanism of apoptotic effects of THC in TM4 Sertoli cells. In accordance, mRNA expression levels of VEGF, EGF, FGF and GDNF were investigated by RT-PCR. Overall, the results indicated a reduction in mRNA levels of all the experimenting growth factors in THC exposed cells (Fig. 3). Accordingly, VEGF and EGF mRNA levels were significantly decreased to 0.47 ± 0.18 and 0.65 ± 0.15, respectively, with a significance value of *P* < 0.05. A more significant reduction was found in FGF and GDNF mRNA levels. Accordingly, FGF mRNA level was significantly decreased to 0.22 ± 0.04 in cells exposed to THC in comparison to the control cells (*P* < 0.001, Fig. 3C). In a similar manner to FGF, there was a significant decrease in GDNF mRNA levels to 0.32 ± 0.1 in cells exposed to THC compared to the control cells (*P* < 0.001, Fig. 3D).

### Effects of THC on protein levels of growth factors in Sertoli cells (western blot assay)

**Fig. 4:**
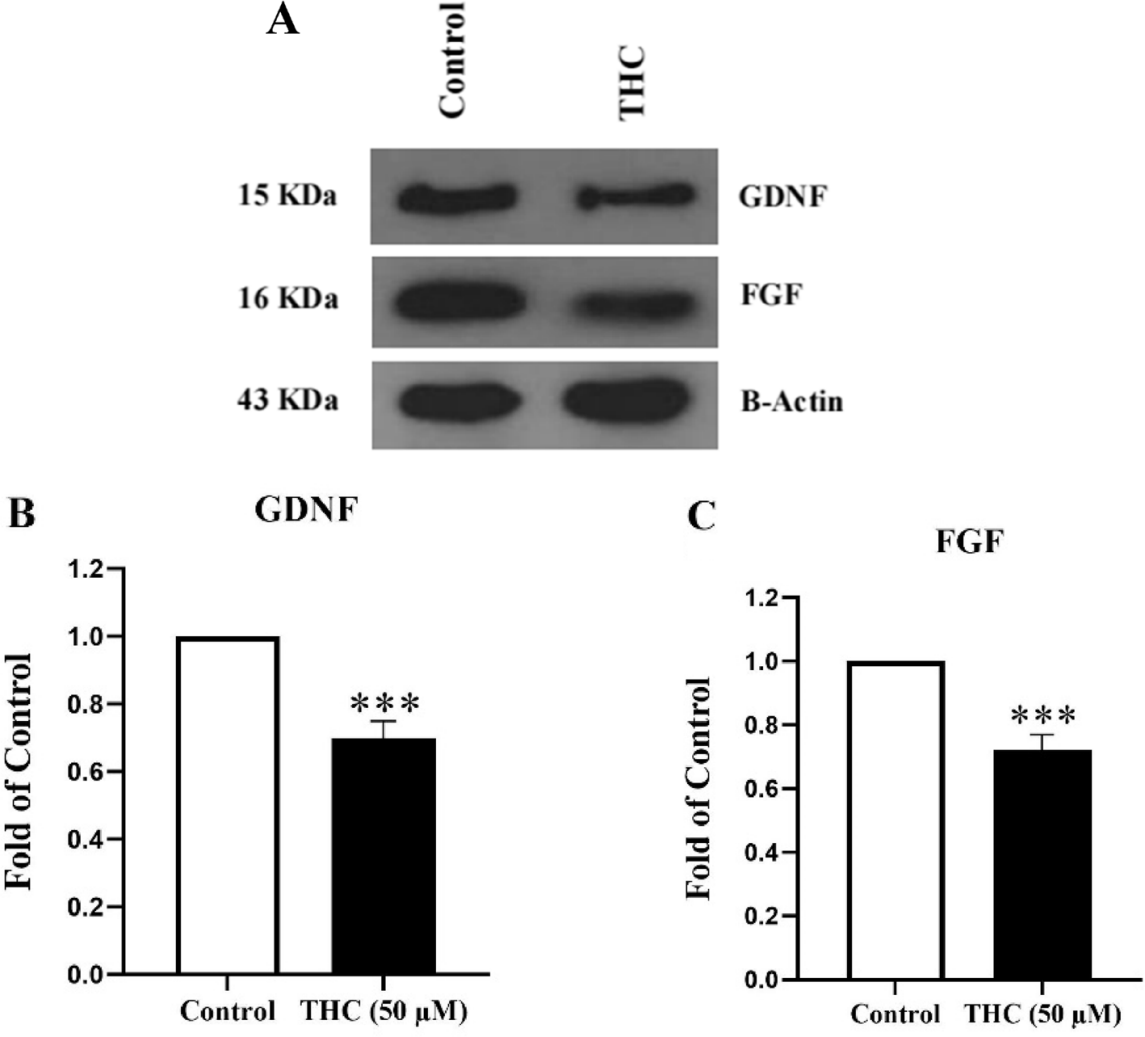
The expression of GDNF and FGF proteins in THC-exposed TM4 cells. **A** Immunoblotting images of expression level of GDNF, FGF, and β-actin proteins. **B **and **C** Bar graphs represent the relative density of each band normalized to that of β-actin as an internal control. Values represent the mean ± SEM of three independent experiments. Stars show the statistical significance of change between the groups. ***p < 0.001 vs. control group (Mann–Whitney U-test)

Following the above mentioned RT-PCR experiments, more significant changes were found in the FGF and GDNF mRNA levels in the THC exposed Sertoli cells. In order to confirm or validate these results, protein expression levels of FGF and GDNF were investigated by western blotting (Fig. 4A-C). GDNF protein level decreased significantly from 1 ± 0.0 in the control group to 0.7 ± 0.05 in THC group (Fig. 4B, *P* < 0.05). FGF protein level was also significantly decreased (0.72 ± 0.05) in the THC exposed Sertoli cells, compared to the control group (Fig. 4C, *P *< 0.05).

## Discussion

In spite of the considerable knowledge about THC-induced testicular toxicity, there is little available information regarding cellular basis and molecular mechanisms underlying this pathological process. Lack of such knowledge interrupts evidence-based development of pharmacological intervention to repair damage caused by THC. On the other hand, no clear correlation between marijuana abuse and reproductive dysfunction can be demonstrated in human studies, since drug-dependent men often abuse other substances such as tobacco, opioids and alcohol as well as marijuana. Moreover, marijuana abuse and its possible correlations to testicular damage and later infertility can usually be studied retrospectively in humans. In vitro models allow the investigation of such relations with prospective, well-controlled study designs and allow for meticulous regulation over experimental conditions, such as the duration and concentration of toxicants exposure. On the other hand, while human and in vivo animal studies can indeed provide valuable insights into the overall impact of toxicants on spermatogenesis, identifying cellular and molecular changes, including the cascade of events, can be challenging when studying the testis as a whole [48]. However, techniques such as sperm staining and RNA and protein extraction from testicular tissue for a range of different cellular and molecular investigation, can indeed provide valuable information in this regard. Therefore, while acknowledging the complementary role of animal experiments, we also emphasize the significance of in vitro models in offering new insights into the study of spermatogenesis, as they provide a practical approach to investigate the cellular and molecular mechanisms underlying testicular injury caused by toxicants [48, 49]. Previous researches show that the detrimental effects of environmental toxins on Sertoli cells, as observed in in vitro models, can be replicated in in vivo studies [23]. The TM4 cell line is the most extensively researched Sertoli cell line and provides a readily available supply of cells with consistent and predictable properties and similar behavior to primary cultures of Sertoli cells [50]. TM4 cells retained Sertoli cell-like characteristics, making them a valuable in vitro model for studying the effects of toxicants such as THC on Sertoli cells [48]. Therefore, the present in vitro study was carried out to investigate cell viability and expression profile of caspase-3 and a number of key testicular growth factors in TM4 Sertoli cells exposed to THC to gain mechanistic insight into the THC-induced testicular toxicity. The current study consisted of two distinct phases. During first phase of the study, we conducted preliminary experiments to explore the impact of THC on Sertoli cell viability and apoptosis. Although this phase was not the primary focus of our research, it served two key objectives. Firstly, it allowed us to validate and confirm prior research on the effects of THC on Sertoli cells viability. Secondly, it enabled us to determine the effective concentration of THC (IC25 or IC50) required for the subsequent phase of our investigation which can vary slightly across different research groups and laboratory conditions. The examination of cell viability using MTT assay and caspase 3 expression by real-time PCR demonstrated that THC reduces cell survival and exhibits a pro-apoptotic effect on isolated TM4 Sertoli cells. THC concentration-dependently decreased the TM4 viability with a significant effect starting at concentration of 1 μM and reached about 75% of the control level at the concentration of 50 μM (IC_25_). The reduction of cell viability indicated by MTT was further confirmed by caspase-3 evaluation. In concordance with our MTT results, there was a significant increase in caspase-3 mRNA expression level in 50 μM THC exposed cells. These findings confirmed our previous results which demonstrated that THC significantly reduced the expression level of pro-caspase3 protein, while simultaneously increasing TUNEL positive apoptotic cells and the expression level of cleaved caspase3 protein in cultured TM4 Sertoli cells [23, 42]. This pro-apoptotic effect of THC was in line with the results of other studies that showed THC, at concentrations comparable to those used in this study, inhibits the proliferation of different cells and enhances apoptosis in different tissues [51–54]. For example, Almadaa et al. (2020), showed that THC at concentration of 15 μM caused a significant decrease in cell viability and activated apoptosis in part through caspase-3 mediated pathways in a human placental cytotrophoblast cell line [54]. In the present study the minimum concentration of THC to show a significant reduction of Sertoli cell viability was 1 μM (Fig. 1), correspond to about 314 ng/ml in man. A blood THC concentration of 314 ng/ml correspond to about 8 times higher than maximum THC blood level in marijuana smokers. According to the literature, the whole-blood THC levels in marijuana smokers or medical cannabis users ranged from 0 to 37 ng/ml depending on the time of testing, marijuana cigarette dose (low or high dose), frequency of smoking (occasionally or frequently) and routes of administration (oral or inhalation) [55–57]. Therefore, what 37 ng/ml means in terms of maximum THC blood level is hard to calculate, as THC levels in the blood peak quickly following consumption then decrease rapidly, making it almost impossible to extrapolate backwards from the concentration of THC at the time of the blood test to the concentration at the time of the consumption [55]. Therefore, based on these explanations and given THC complex pharmacokinetics, indicating precise evidence-based blood levels for THC is challenging [58]. On the other hand, a clear comparison between the concentration level of THC in human, in vivo and in vitro studies is not possible, since the in vitro experiments performed at non-physiological conditions do not necessarily correspond to in vivo results. All together, these data indicate that threshold concentration of THC to show a significant reduction of Sertoli cell viability seems to be higher than average THC blood level in marijuana users. However we have several explanations to claim that regular and prolonged cannabis consumers are at risk of Sertoli cells damage. Firstly, the oral administration of THC produces more active metabolite, which could more efficiently reach the effect site than THC [59–61]. Secondly, the slow absorption kinetics of THC produces sustained plateau levels in the blood, which could influence the body and tissue distribution. Giroud et al. found that, in a cocaine fatality, THC and its metabolites were in higher concentration in brain than in blood [62]. Thirdly, because cannabinoids accumulate in fat, chronic marijuana consumption may induce more blood THC level after a week or more of abstinence [58]. And finally, we have recently shown that prolonged exposure to lower concentrations of THC led to significant reduction of Sertoli cell viability in an in vitro model [23]. Accordingly, exposure to 0.01 and 0.1 μM THC (correspond to 3.14 and 31.4 ng/ml, respectively, in man) for a period of 48 hours caused a significant reduction in TM4 survivability.

THC exposure may interfere with Sertoli cell function, resulting in abnormalities in Sertoli cell markers. In the second phase of our study, which was the main objective of our study, we aimed to gain a deeper understanding of the mechanisms underlying THC-induced testicular toxicity, with a specific emphasis on THC-induced Sertoli cell apoptosis. To address this gap and using real-time RT-PCR, we demonstrated that THC at the concentration determined from the first phase of our study (IC_25_) decreased mRNA and protein expression level of a number of key testicular growth factors. Previous researches have shown that the influence of the cannabinoid system on the expression or production of growth factors in both normal cells (with controlled proliferation activity) and tumor cells (with poor differentiation and previously enhanced proliferation activity) is subject to variation based on differences in experimental conditions. Canabinoid system can lead to different and paradoxical effects on growth factors depending on the type of cell and tissue, canabinoid receptor subtypes, expression/localization patterns, affinity, as well as the concentration and duration of exposure to cannabinoids. Multiple growth factors are likely to regulate Sertoli cells proliferation in vivo, with a key role for VEGF, EGF, FGF and GDNF in testicular cell proliferation [26–30]. According to previous reports, cannabis treatment decreased secreted protein and mRNA expression level of VEGF in prostate cancer cell lines [63]. Moreover, THC (10–100 μM) inhibited the proliferation and expression of EGF receptors [64] and reduced production of EGFs [39] in lung cancer cells. In non-cancer cells, THC (20 μM) caused a reduction in the production of insulin-like growth factor 2 in human placental BeWo cells [43]. It has also been reported that high concentrations of cannabidiol decreased transforming growth factor (TGF)b production in human fibroblast extracellular matrix [65]. Moreover, increased plasma levels of cannabinoids were associated with lower VEGF concentrations in medical cannabis users among chronic pain patients [66] and with lower circulating levels of brain-derived neurotrophic factor [67] and nerve growth factor [68] in physically active cannabis users. Our findings were in agreement with the above mentioned studies and showed that THC, at concentrations comparable to those used in these studies, significantly down-regulated the mRNA expression levels of VEGF (by as much as 53%), EGF (35%), FGF (78%) and GDNF (68%) in TM4 Sertoli cells. The decrease of FGF and GDNF mRNA levels indicated by RT-PCR was further confirmed by western blotting assay. In concordance, THC exposure resulted in down-regulation of FGF (28%) and GDNF (30%) protein levels. These findings suggested that THC shows cytotoxic and apoptotic effects in TM4 Sertoli cells partly through down-regulation of growth factors expression. If caused in vivo, this may manifest as increased testicular apoptosis and hence compromised testicular growth and function in marijuana smokers or medical cannabis users. Such findings suggest that marijuana exposure either recreationally or medicinally may increase the susceptibility to Sertoli cell-based reproductive dysfunction. However, the exact intracellular signaling pathways and molecular mechanisms through which the activation of the cannabinoid system leads to low expression of growth factors and apoptosis are not fully understood and may vary depending on the specific context and cell type involved. Several pathways and factors (cyclooxygenase- and prostaglandin-mediated mechanisms, nitro-oxidative stress and immune-inflammatory signaling pathways) could be implicated in this phenomenon [43, 69]. Accordingly, a recent research indicates that THC caused a reduction in the secretion of insulin growth factor 2 in human trophoblast cells, via oxidative stress responses [43]. Cannabinoids could also potentially affect gene expression, transcription factors or nuclear receptors that regulate the production of growth factors. For example, peroxisome proliferator-activated receptors (PPARs), which are a family of nuclear receptors and regulators of a plethora of target genes involved in cell differentiation, proliferation and growth factors production [70, 71], are activated by a large number of both phyto- and endo-cannabinoids [70]. Likewise, growth factors can affect apoptotic pathways through different signaling mechanisms, depending on the specific type of growth factor and the type of cell being modified. For instance, it has been reported that the activation of PI3k/Akt signaling pathway by growth factors like GDNF and FGFs can trigger the Akt signaling cascade, indirectly promoting the proliferation, differentiation, and protection against cell death of spermatogonial stem cells and Sertoli cells [72]. Moreover, the growth factors have the ability to activate the mitogen-activated protein kinase (MAPK/ERK) pathway, which can override apoptotic signals from death receptors and can also hinder the activation of pro-apoptotic proteins from the BCL-2 family, such as BAX and BIM, while stimulating the expression of anti-apoptotic members like BCL-2, and BCL-XL [73, 74]. It has been reported that THC-induced apoptosis was preceded by significant changes in the expression of genes involved in the MAPK signal transduction pathways in leukemic cell lines [75]. Altogether, we hypothesize that activation of cannabinoid system by THC in TM4 cells may lead to low expression of growth factors which, in turn, triggers apoptotic pathways. Consistent with this hypothesis it has been shown that cannabinoids suppressed the TGF-induced activation of MAPKs in human Tenon’s fibroblasts [76]. THC also inhibited EGF-induced growth by preventing the EGF-induced phosphorylation of ERK1/2, JNK1/2 and AKT in human lung cancer cell lines [39]. However, further molecular and cellular researches and biochemical measurements are needed to fully understand the possible molecular connections and pathways between cannabinoid system, expression of growth factors and apoptotic mechanisms. Conducting research using cultured primary Sertoli cells and adult Sertoli cells, in vivo models of THC administration in animal models, and a seminiferous tubule culture approach could also help elucidate these relationships and the related hypotheses.

In summary, exposure to THC significantly decreased cell viability and increased apoptosis in TM4 Sertoli cells, at least in part, through growth factors-dependent pathways. Decreased cell viability, over-expression of caspase-3 mRNA level and down-expression of growth factors mRNA and protein levels in Sertoli cells exposed to THC may be a reflection of THC-induced testicular injury resulting in enhanced Sertoli cell apoptosis, which may be partly involved in reproductive dysfunction associated with marijuana smokers or medical cannabis users.

### Supplementary Information

**Additional file 1.**

**Additional file 2.**

**Additional file 3.**

## Data Availability

The datasets during and/or analysed during the current study available from the corresponding author on reasonable request.
