# Supplementary figures and images for: In vitro evaluation of cell viability and expression profile of growth factors in mouse Sertoli cells exposed to Delta-9-tetrahydrocannabinol: a mechanistic insight into the cannabinoid-induced testicular toxicity
Source: BMC Pharmacol Toxicol. 2023 Nov 9;24:61. doi: 10.1186/s40360-023-00704-8 (PMC10636832; doi:10.1186/s40360-023-00704-8)

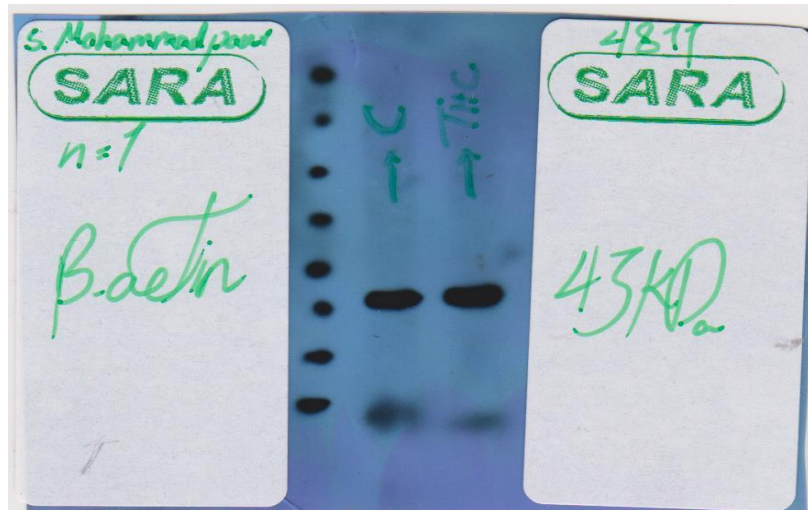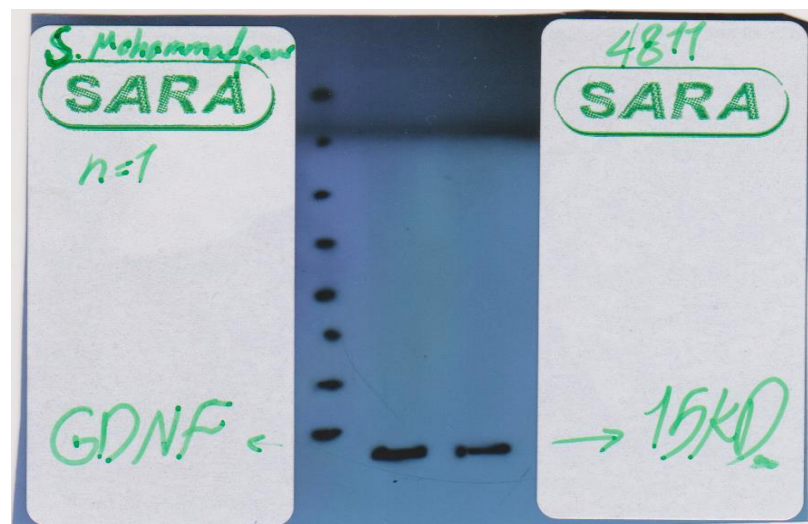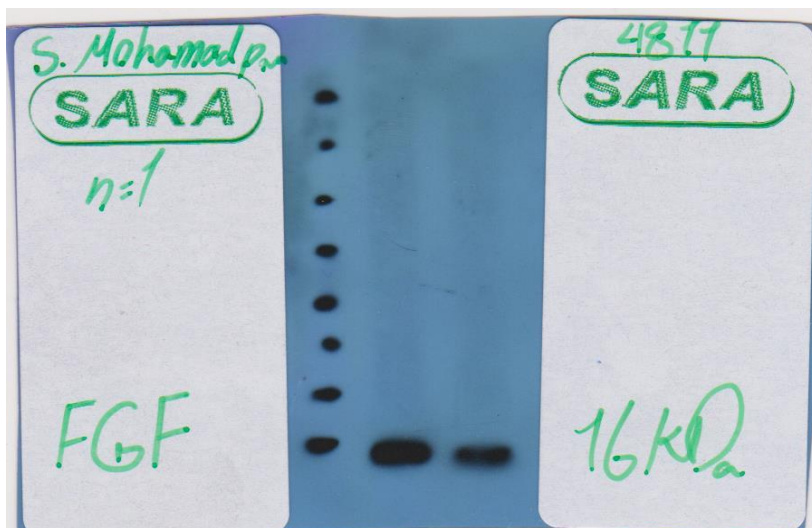

Supplement: Supplementary file 1 — Additional file 1. [file 40360_2023_704_MOESM1_ESM.pdf]

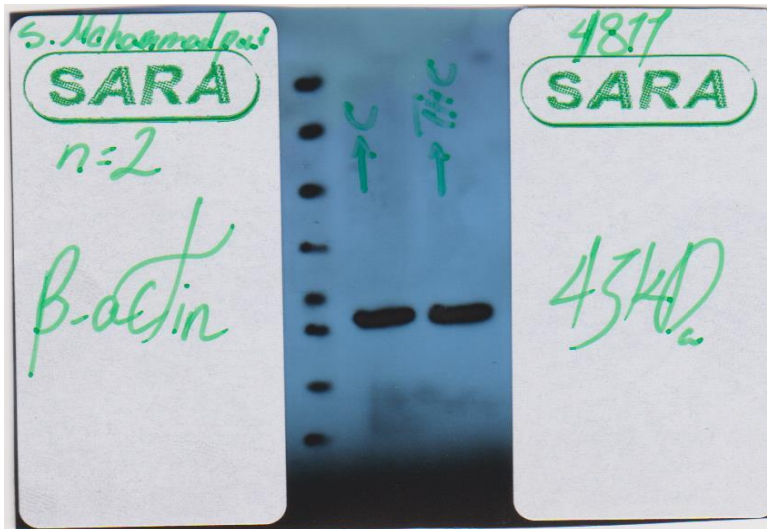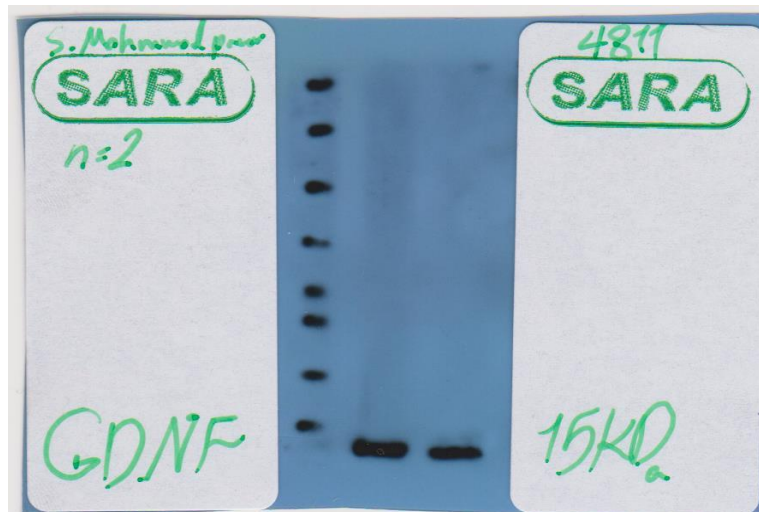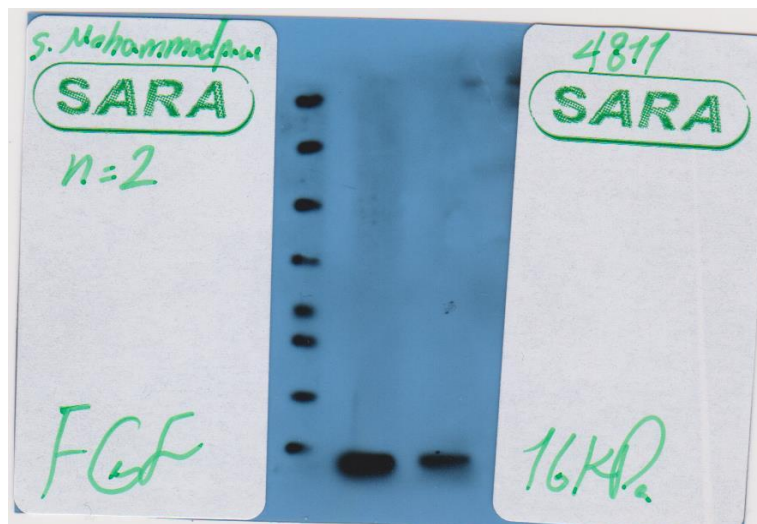

Supplement: Supplementary file 2 — Additional file 2. [file 40360_2023_704_MOESM2_ESM.pdf]

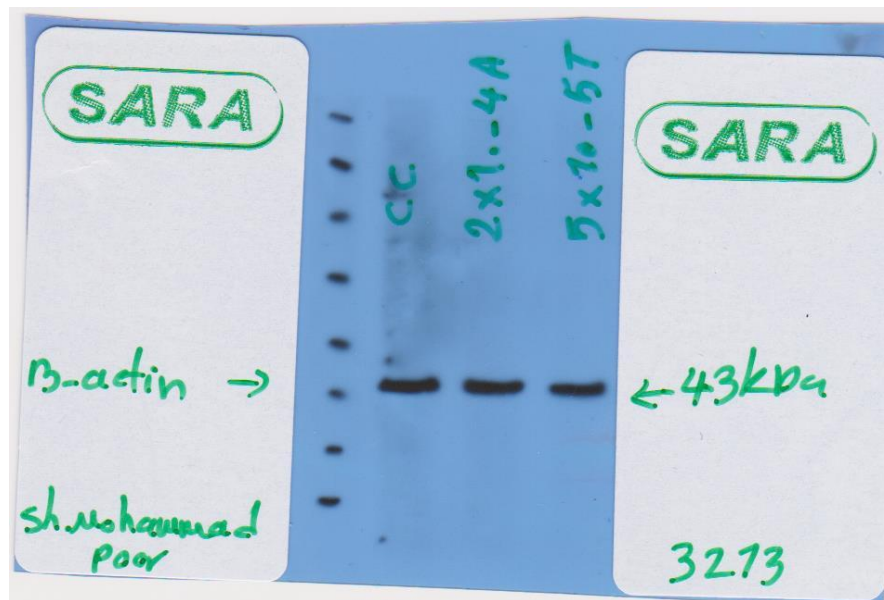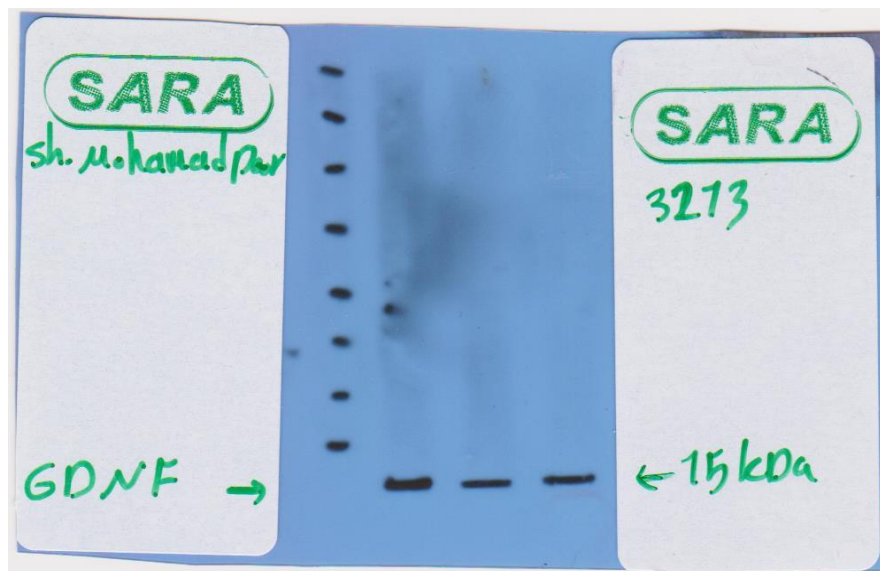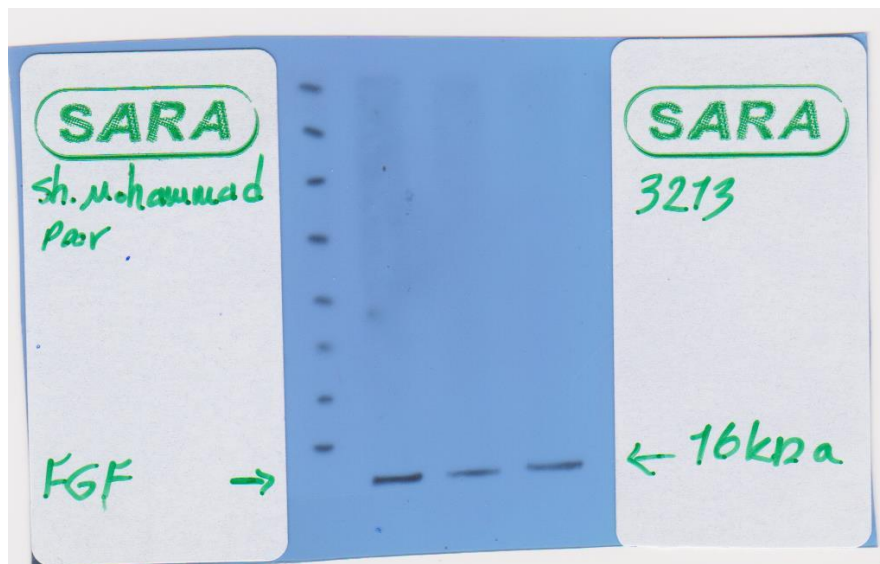

Supplement: Supplementary file 3 — Additional file 3. [file 40360_2023_704_MOESM3_ESM.pdf]
